# Diagnosis and Management of Cervical Vestigial Pathologies in Children: A Retrospective Study of 25 Cases in the Drâa-Tafilalet Region of Morocco

**DOI:** 10.7759/cureus.112530

**Published:** 2026-07-12

**Authors:** Abdelouahid Taleuan, Adnane Lakhal, Mohammed ElAmine Squalli Houssaini, Abdellatif Oudidi

**Affiliations:** 1 Department of Otolaryngology-Head and Neck Surgery, Al Amir Sultan Hospital, Errachidia, MAR; 2 Department of Otolaryngology-Head and Neck Surgery, Centre Hospitalier Universitaire (CHU) Hassan II, Fez, MAR

**Keywords:** branchial cleft anomalies, cervical congenital lesions, pediatric neck mass, sistrunk procedure, thyroglossal duct cyst

## Abstract

Background

Cervical vestigial pathologies are common congenital anomalies in children, arising from embryological remnants of the branchial apparatus and the thyroglossal duct. They constitute an important etiology of cervical masses in pediatric otolaryngology practice.

Objective

This study aimed to characterize the epidemiological, clinical, and therapeutic specificities of cervical vestigial pathologies in children in the Drâa-Tafilalet region of Morocco and to compare our findings with data from the literature.

Methods

We conducted a retrospective descriptive study over a one-year period (January-December 2025) in the Department of Otolaryngology-Head and Neck Surgery at Al Amir Sultan Hospital, Errachidia, Morocco. A total of 25 pediatric patients diagnosed with cervical vestigial pathologies were included. Epidemiological, clinical, radiological, therapeutic, and histopathological data were collected and analyzed.

Results

The mean age at diagnosis was 6.8 years (range: 2-16 years), with a slight male predominance (13 males, 12 females; sex ratio: 1.08). Cervical swelling was the most frequent presenting symptom. Thirteen patients presented with a cervical mass (10 midline, three lateral). The initial presentation was infectious in six cases, fistulization in five cases, and compressive symptoms in one case. Thyroglossal duct cysts were the most common lesions (12 cases, 48%), followed by second branchial cleft cysts (five cases, 20%), thyroglossal duct fistulas (three cases, 12%), dermoid cysts (three cases, 12%), and cystic lymphangiomas (two cases, 8%). Ultrasound was performed in all patients; computed tomography (CT) was required in two cases. All patients underwent surgical excision. The Sistrunk procedure was used for thyroglossal duct lesions; complete excision of the fistulous tract was performed for branchial anomalies. Histopathological examination confirmed the diagnosis in all cases. No postoperative complications were recorded. Recurrence occurred in one patient (4%) and was successfully managed by revision surgery.

Conclusion

Cervical vestigial pathologies in children are predominantly benign but require accurate diagnosis and complete surgical excision to prevent recurrence. The Sistrunk procedure and complete fistulous tract excision remain the gold standard treatments in this setting.

## Introduction

Cervical vestigial anomalies are congenital lesions resulting from the incomplete involution of embryological structures, particularly the branchial apparatus and the thyroglossal duct. They represent one of the most common causes of neck masses in the pediatric population [1-3].

These lesions typically present as midline or lateral cervical swellings and may be complicated by infection, fistulization, or compressive symptoms. The most frequent entities include thyroglossal duct cysts, branchial cleft anomalies, dermoid cysts, and lymphatic malformations [2,4].

The aim of this study was to describe the epidemiological, clinical, radiological, therapeutic, and outcome characteristics of cervical vestigial pathologies in children managed at our institution, to highlight the specificities of these lesions in the Drâa-Tafilalet region, a resource-limited and geographically isolated area of southern Morocco with limited published epidemiological data, and to compare our findings with those reported in the current literature.

## Materials and methods

This retrospective descriptive study was conducted over a one-year inclusion period (January 1-December 31, 2025) in the Department of Otolaryngology-Head and Neck Surgery at Al Amir Sultan Hospital, Errachidia, Morocco. The dataset was locked on May 31, 2026. Patients were followed until May 2026, yielding a maximum follow-up duration of 16 months for patients included at the beginning of the study period and a minimum of five months for those included at the end.

Study population

Pediatric patients under 18 years of age, diagnosed with cervical vestigial pathologies during the study period, were included. Diagnosis was based on a combination of clinical examination, radiological findings, and histopathological confirmation.

Data collection

Medical records were reviewed, and the following variables were collected: age and sex, clinical presentation (type and duration of symptoms), cervical lesion location (midline or lateral), imaging findings, type of surgical procedure performed, histopathological results, and postoperative outcomes, including complications and recurrence.

Imaging evaluation

All patients underwent cervical ultrasound as the first-line imaging modality. Computed tomography (CT) was performed in two cases for further evaluation of deep or anatomically complex lesions. No magnetic resonance imaging (MRI) was performed during the study period.

Treatment protocol

All patients underwent surgical management after appropriate preoperative preparation. In cases presenting with active infection, antibiotic therapy was initiated, and surgery was deferred until the resolution of the acute inflammatory phase. Thyroglossal duct cysts and fistulas were treated using the Sistrunk procedure. Branchial cleft anomalies were managed by the complete surgical excision of the cyst or fistulous tract. Dermoid cysts and lymphatic malformations were treated by complete excision when technically feasible.

Histopathological analysis

All surgical specimens were systematically submitted for histopathological examination to confirm the diagnosis.

Ethical considerations

This study was conducted in accordance with institutional ethical standards. Patient confidentiality was strictly maintained, and no personal identifiers were disclosed in the analysis. As a retrospective observational study, formal ethics committee approval was not required per institutional policy.

Statistical analysis

Data were analyzed using descriptive statistics. Categorical variables were expressed as frequencies and percentages, and continuous variables were expressed as means with standard deviations and ranges. All statistical analyses were performed using Microsoft Excel 2019 (Microsoft Corp., Redmond, WA, USA).

## Results

Epidemiological data

A total of 25 pediatric patients were included. The mean age at diagnosis was 6.8 years (range: 2-16 years). There was a slight male predominance, with 13 males (52%) and 12 females (48%), yielding a sex ratio of 1.08.

Clinical presentation

**Figure 1:**
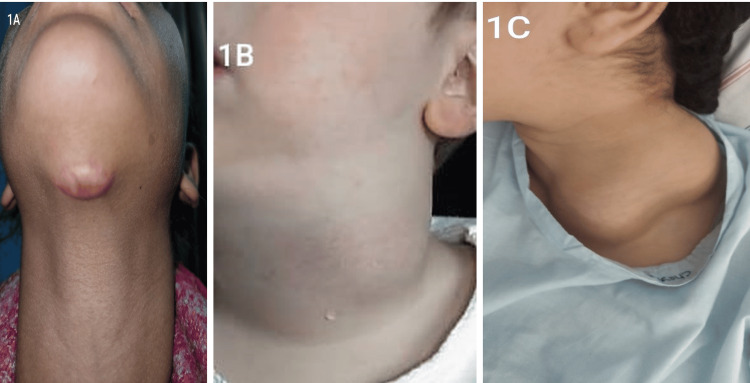
Clinical appearance of cervical vestigial lesions. (A) Thyroglossal duct cyst presenting as a well-defined midline cervical swelling. (B) Second branchial cleft cyst presenting as a lateral cervical mass located at the mandibular angle, along the anterior border of the sternocleidomastoid muscle. (C) Cystic lymphangioma presenting as a large cystic lesion involving the lateral and posterior cervical regions.

Cervical swelling was the most common presenting symptom. Thirteen patients initially presented with a cervical mass, of which 10 were midline and three were lateral (Figure 1).

The mode of presentation varied among patients: an infectious presentation was observed in six cases (24%), characterized by painful swelling with local inflammatory signs; fistulization was noted in five patients (20%), with the lesion revealed by cutaneous discharge; and compressive symptoms (dyspnea and dysphagia) were reported in one patient (4%).

Lesion distribution

The distribution of lesions is summarized in Table 1.

**Table 1:** Distribution of cervical vestigial pathologies in the study population (n = 25).

Pathology	n	%
Thyroglossal duct cysts	12	48%
Second branchial cleft cysts	5	20%
Thyroglossal duct fistulas	3	12%
Dermoid cysts	3	12%
Cystic lymphangiomas	2	8%
Total	25	100%

The clinical characteristics of all patients are detailed in Table 2.

**Table 2:** Clinical characteristics of the 25 patients included in the study. *Recurrence at 12 months, managed by revision surgery. ^†^Initially suspected KTG; histopathology confirmed a dermoid cyst. KTG: thyroglossal duct cyst; FTG: thyroglossal duct fistula; KB2: second branchial cleft cyst; KD: dermoid cyst; LK: cystic lymphangioma; AB: antibiotic therapy; US: ultrasound; CT: computed tomography

#	Age (yrs)	Sex	Diagnosis	Presentation	Location	Imaging	Treatment	Complications	Recurrence	Follow-up (mo)
1	4	M	KTG	Cervical swelling	Midline	US	Sistrunk procedure	None	No	12
2	6	F	KTG	Cervical swelling	Midline	US	Sistrunk procedure	None	No	10
3	3	M	KTG	Infectious	Midline	US	AB + Sistrunk procedure	None	No	14
4	8	F	KTG	Cervical swelling	Midline	US	Sistrunk procedure	None	No	9
5	5	M	KTG	Infectious	Midline	US	AB + Sistrunk procedure	None	No	11
6	7	F	KTG	Cervical swelling	Midline	US	Sistrunk procedure	None	No	8
7	2	M	KTG	Cervical swelling	Midline	US	Sistrunk procedure	None	No	10
8	9	F	KTG	Infectious	Midline	US	AB + Sistrunk procedure	None	No	16
9	11	M	KTG	Cervical swelling	Midline	US	Sistrunk procedure	None	Yes*	15
10	6	F	KTG	Cervical swelling	Midline	US	Sistrunk procedure	None	No	9
11	4	M	KTG	Fistulization	Midline	US	Sistrunk procedure	None	No	7
12	5	F	KTG	Cervical swelling	Midline	US	Sistrunk procedure	None	No	5
13	8	M	KB2	Infectious	Lateral	US	AB + complete excision	None	No	12
14	12	M	KB2	Infectious	Lateral	US	AB + complete excision	None	No	10
15	10	F	KB2	Cervical swelling	Lateral	US	MacFee + complete excision	None	No	8
16	7	F	KB2	Fistulization	Lateral	US	Complete fistula excision	None	No	11
17	9	M	KB2	Fistulization	Lateral	US	Complete fistula excision	None	No	9
18	6	F	FTG	Fistulization	Midline	US	Sistrunk procedure	None	No	10
19	4	M	FTG	Fistulization	Midline	US	Sistrunk procedure	None	No	8
20	5	F	FTG	Infectious	Midline	US	AB + Sistrunk procedure	None	No	12
21	3	M	KD	Cervical swelling	Midline	US	Complete excision	None	No	7
22	7	F	KD	Cervical swelling	Midline	US	Complete excision	None	No	6
23	5	M	KD^†^	Cervical swelling	Midline	US	Complete excision	None	No	9
24	16	M	LK	Compressive	Lateral	US + CT	Complete excision	None	No	14
25	8	F	LK	Cervical swelling	Lateral	US + CT	Complete excision	None	No	10

Imaging findings

**Figure 2:**
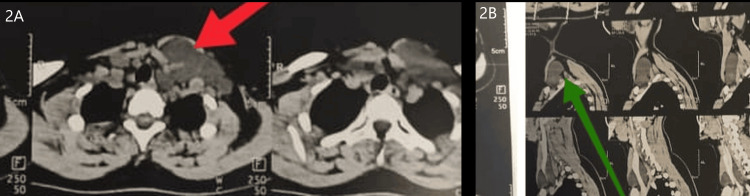
Contrast-enhanced cervicothoracic CT scan of a pediatric patient with cystic lymphangioma. (A) Axial images: the red arrow indicates a large multiloculated hypodense cystic lesion in the left lateral cervical region, well-circumscribed, with mild mass effect on adjacent cervical structures and no solid enhancing components. (B) Sagittal images: the green arrow demonstrates the cervicothoracic extension of the lesion, highlighting its craniocaudal spread across the cervical and superior mediastinal compartments. The overall imaging pattern is consistent with cystic lymphangioma. Digital DICOM images were not available for this case due to limitations in archiving infrastructure at our institution. The CT images presented represent the best available documentation of this case. CT: computed tomography; DICOM: Digital Imaging and Communications in Medicine

Cervical ultrasound was performed in all 25 patients as the first-line imaging modality. It confirmed the cystic nature of the lesions and their anatomical location in all cases. In cases of thyroglossal duct cyst, ultrasound also confirmed the presence and normal position of the thyroid gland, which is essential prior to surgical intervention to rule out an ectopic thyroid. CT scan was performed in two cases to assess lesion extension in complex presentations (Figure 2).

MRI was not performed in our series, owing to limited local availability and associated cost constraints.

Surgical treatment

All 25 patients underwent surgical management. In cases presenting with active infection, surgery was performed after the resolution of the acute inflammatory phase following appropriate antibiotic therapy.

**Figure 3:**
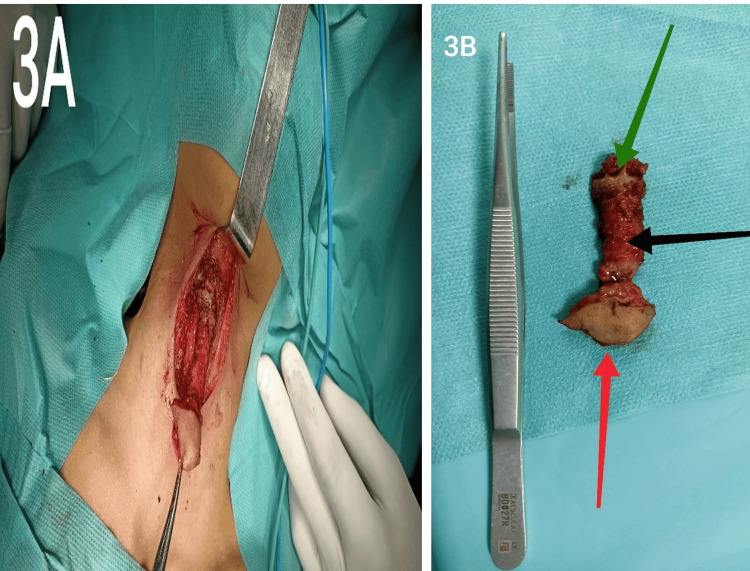
Intraoperative photographs of the Sistrunk procedure for thyroglossal duct cyst excision. (A) Intraoperative view showing the surgical field during the en bloc dissection of the cervical midline structures. (B) Surgical specimen after complete excision: the red arrow indicates the thyroglossal duct cyst, the black arrow indicates the thyroglossal tract, and the green arrow indicates the central portion of the hyoid bone. The specimen illustrates the en bloc resection including the cyst, the tract, and the hyoid bone segment, performed to minimize the risk of recurrence.

Thyroglossal duct cysts and fistulas were treated using the Sistrunk procedure, which included the en bloc excision of the cyst or fistulous tract along with the central portion of the hyoid bone and the suprahyoid tissue tract (Figure 3).

**Figure 4:**
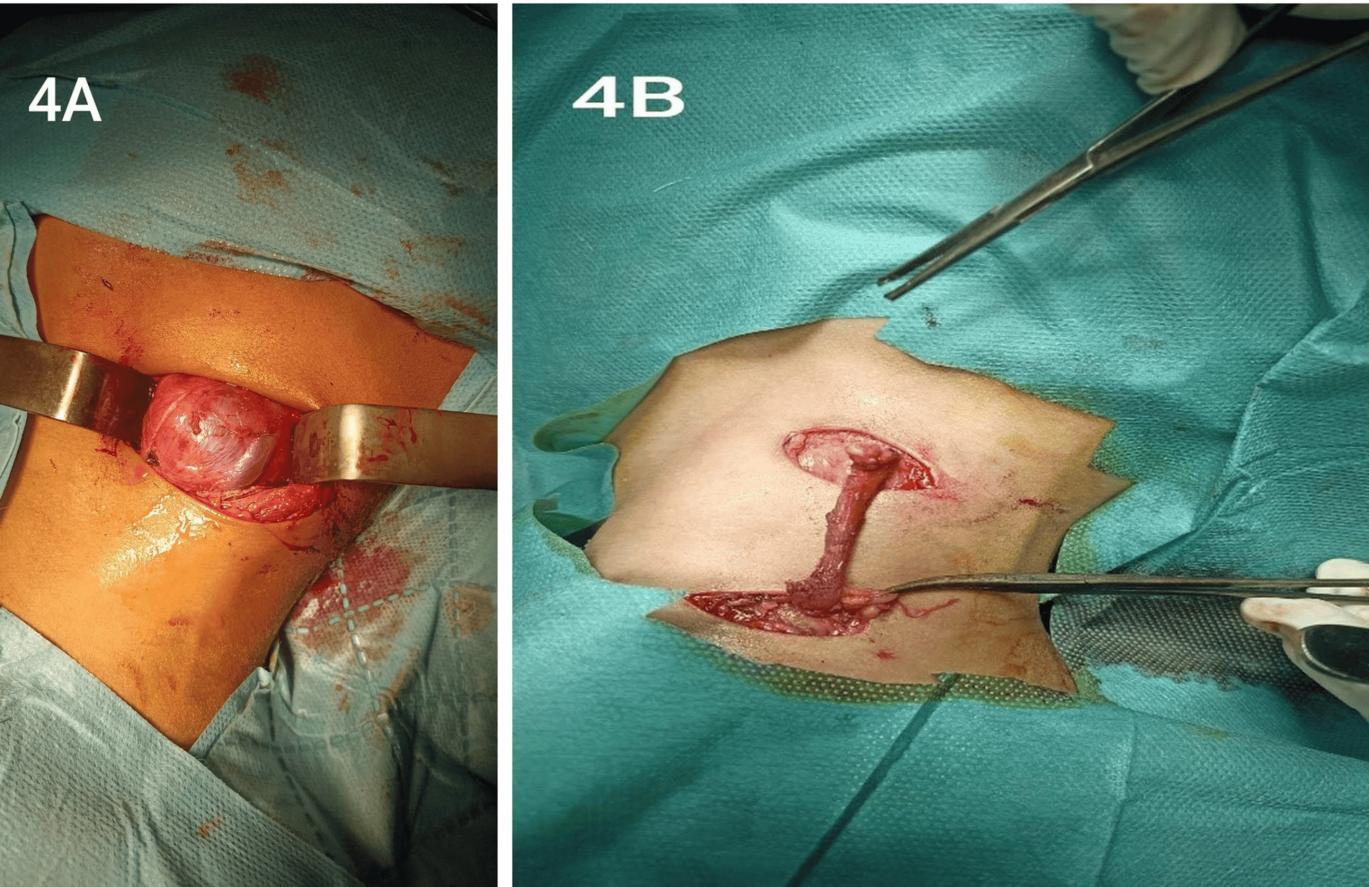
Intraoperative photographs of the surgical management of a second branchial cleft cyst. (A) Standard lateral cervical incision used for the excision of the cystic lesion. (B) MacFee double-incision approach used in a large second branchial cleft cyst to improve surgical exposure and ensure complete excision.

Second branchial cleft cysts were managed by complete surgical excision. In one case, a MacFee (two-transverse-incision) approach was used to facilitate adequate exposure and ensure the complete removal of a large lateral cervical cyst (Figure 4).

**Figure 5:**
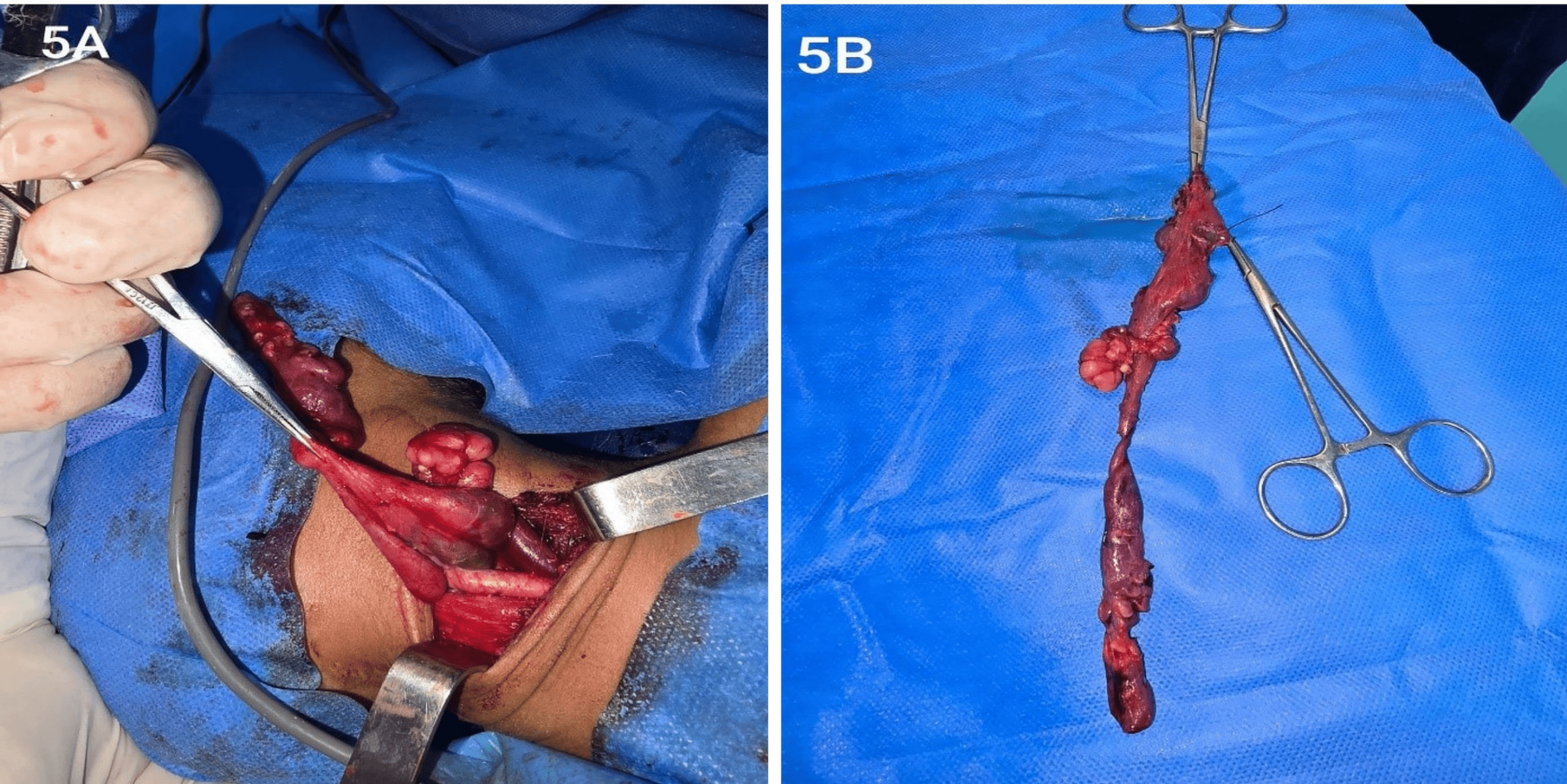
Intraoperative photographs of cystic lymphangioma excision. (A-B) Careful dissection and complete surgical excision of the cystic lesion with the preservation of adjacent cervical vascular and nervous structures.

Dermoid cysts were treated by complete excision, as were the cystic lymphangiomas, with careful dissection to preserve adjacent neurovascular structures. Complete excision was achieved in all patients (Figure 5).

Histopathological findings

Histopathological examination was performed in all 25 patients and confirmed the clinical diagnosis in every case. Thyroglossal duct cysts showed a pseudostratified ciliated columnar epithelial lining with surrounding thyroid follicular tissue. Branchial cleft cysts demonstrated stratified squamous epithelium with prominent lymphoid follicles in the cyst wall. Dermoid cysts were lined by keratinizing squamous epithelium with adnexal skin appendages including hair follicles and sebaceous glands. Cystic lymphangiomas showed thin-walled channels lined by flat endothelial cells with surrounding lymphoid tissue. Notably, one lesion initially suspected to be a thyroglossal duct cyst based on clinical and intraoperative findings was ultimately diagnosed as a dermoid cyst on final pathological analysis, highlighting the importance of systematic histopathological examination in all cases. Representative histopathological images could not be included due to the absence of digital pathology infrastructure at our institution, which represents a recognized limitation of this study and has been explicitly acknowledged in the Limitations section.

Postoperative outcomes and follow-up

No postoperative complications were recorded in our series. Patients were followed up for a mean duration of 10 months (range: 5-16 months). Recurrence was observed in one patient (4%), involving a thyroglossal duct cyst. This patient underwent successful revision surgery with no further recurrence during follow-up.

## Discussion

Cervical vestigial pathologies are among the most frequent congenital causes of neck masses in children, originating from embryological remnants of the branchial apparatus and the thyroglossal duct [1,2]. In our series, thyroglossal duct cysts were the most common lesions (48%), followed by branchial cleft anomalies, a distribution consistent with large pediatric series reported in the literature [3,4]. Recent studies further confirm this epidemiological pattern, reinforcing the predominance of thyroglossal duct cysts in pediatric populations worldwide [5,6].

The mean age at diagnosis in our study (6.8 years) is consistent with published literature reporting early childhood presentation in most cases [2,7]. A slight male predominance was observed in our cohort, although sex distribution remains inconsistent across published series, with some studies reporting equal sex distribution [4,8,6]. Muhialdeen et al. also confirmed a similar age distribution in their series of thyroglossal duct cysts [5].

Clinically, cervical swelling is the most common presenting symptom, as observed in our series [1]. Infectious complications and fistulization are frequently reported in delayed presentations and in branchial anomalies [7,5]. These complications were similarly represented in our cohort, reflecting the well-described clinical behavior of these lesions. The 24% rate of infectious presentation in our series is comparable to rates reported in the literature, ranging from 20% to 30% [5,7].

Regarding imaging, ultrasound remains the first-line diagnostic modality due to its wide accessibility, absence of ionizing radiation, and high diagnostic reliability for superficial cystic lesions [9]. It was performed in all 25 patients in our series and confirmed the diagnosis in every case. CT scan is reserved for anatomically complex cases or when deep extension is suspected [10], as was the case in our two patients with cystic lymphangioma. MRI is recommended for deep or extensive lesions; however, it was not performed in our series due to limited local availability and cost constraints, which is a recognized challenge in resource-limited settings [7].

Surgical excision remains the cornerstone of treatment for all cervical vestigial pathologies. The Sistrunk procedure is considered the gold standard for thyroglossal duct cysts, incorporating the en bloc excision of the cyst, the thyroglossal tract, and the central portion of the hyoid bone, which significantly reduces recurrence rates compared to simple cyst excision alone [1,3,11]. This technique was applied in all thyroglossal duct lesions in our series. A review by Righini et al. emphasizes that strict adherence to the Sistrunk technique is the primary determinant of recurrence prevention [12].

Complete excision of branchial cleft anomalies, including the entire fistulous tract when present, is essential to prevent recurrence [7,13]. In our series, one patient with a large second branchial cleft cyst required a MacFee double-incision approach to ensure complete excision, consistent with recommendations in the literature for anatomically complex cases. Novel minimally invasive approaches, including endoscopic techniques, have been described for selected cases [14], although these were not available at our institution.

Histopathological examination confirmed the clinical diagnosis in all patients and provided important diagnostic information. The finding of one lesion initially suspected to be a thyroglossal duct cyst that was ultimately confirmed as a dermoid cyst on histopathology highlights the importance of systematic pathological examination of all surgical specimens, as previously reported in the literature [5]. Representative histopathological images could not be included due to the absence of digital pathology infrastructure at our institution, which has been acknowledged in the Limitations section.

The recurrence rate in our study was 4% (one case), involving a thyroglossal duct cyst successfully managed by revision surgery. This rate compares favorably with published data, where recurrence after the Sistrunk procedure ranges from 3% to 15% depending on the completeness of surgical excision and the surgeon's experience [11,12]. Dimock et al. recently reported a case of thyroglossal duct cyst recurrence with multiple postoperative complications, emphasizing the technical challenges of revision surgery in pediatric patients [15].

This study contributes data from a resource-limited, geographically isolated region of southern Morocco where published epidemiological data on congenital cervical pathologies in children remain scarce. The results demonstrate that satisfactory surgical outcomes can be achieved with standard techniques even in settings with limited diagnostic infrastructure, provided that appropriate patient selection and careful surgical planning are ensured [7,11].

Limitations

The main limitations of this study include its retrospective design, the relatively small sample size inherent to the single-center one-year study period, the absence of MRI in complex cases, and the unavailability of digital histopathological imaging due to limited pathology infrastructure at our institution. Prospective multicenter studies with longer follow-up would provide stronger evidence and more detailed outcome data.

## Conclusions

Cervical vestigial pathologies in children are predominantly benign congenital lesions with variable clinical presentations. Accurate diagnosis based on clinical assessment and appropriate imaging, followed by complete surgical excision, is essential to ensure optimal outcomes and minimize the risk of recurrence. The Sistrunk procedure for thyroglossal duct lesions and the complete excision of branchial fistulous tracts remain the gold standard surgical techniques in this context.
